# Correspondence

**Published:** 1884-07

**Authors:** H. F. James

**Affiliations:** (Ontario), 2627 Olive Street, St. Louis, Mo.


### CORRESPONDENCE.

*To the Editor Journal Comparative Medicine and Surgery:*

A few weeks ago a Mr. Smith, representing the "U. S. Veterinary Journal," published at Chicago, Ill., called on the graduates of this city for the purpose, as he said, of initiating a movement to form a State Association for Missouri, and thus furthering the interests of the profession. The call for a convention, which he showed us, was signed by half a dozen notorious quacks, not one graduate name being on the list. He asked for our signature individually, but each one, wishing to consult with the others, requested him to call again, though on account of its commercial savor and the questionable signatures, we felt inclined to fight shy of the whole affair. This gentleman was finally referred to me by two or three of my colleagues, they agreeing to stand by my decision. The other graduates held their signatures in reserve, presumably to see how we acted. I told Mr. Smith that on no consideration would we recognize the quacks; that the way to elevate the profession in public estimation was not to call a convention of graduates of the stable fork and broom, and trumpet forth such a proceeding as in the interests of veterinary science. No compromise could be effected, not even to the extent of admitting those men who had practised ten years or more. The line must be drawn somewhere, and we would have nothing to do with any association which was not formed exclusively of graduates of recognized colleges. At any rate, we did not consider that the time was yet ripe for the formation of a State Association; nearly all the graduates in Missouri had recently settled down, and could ill afford to leave their newborn practice at the present time. I pointed out the danger of calling an indiscriminate convention of practitioners; how the quacks could outvote us ten to one, and have everything their own way. To this Mr. Smith replied that their great idea was to advance our interests, and as a secondary matter, secure the position of the "U. S. Veterinary Journal" as the official organ of the Association. That the other State Associations were so largely tainted with the empirical element (a fact which, I forgot to state, I asked him about in our conversation), was their own fault; the "Journal" only asked those who were reputed to be graduates to sign these convention calls, and it remained with ourselves to examine the credentials of such as presented themselves for admission. He was sorry to find we were not in favor of the convention, and hoped there would be a more favorable outlook in the future. Mr. Daniels would have made arrangements with the railways and hotels for reduced rates to those who desired to attend. If the graduates would not take the matter up, of course the whole thing would have to fall through. Before he left, I told Mr. Smith that the profession in St. Louis would be found ready at any time to advance the cause of science, but we could not possibly consent to recognize these proposed constituents of the Association as our equals, and advised him in future to get a list of the graduates of what recognized colleges there are or have been in existence, and exercise a little more circumspection as to the qualifications of those persons who have been signing these calls for conventions in the various States. We flatter ourselves that Mr. S. found the graduates of St. Louis entertained a higher idea of both professional duty and dignity than was shown by some of the regulars in other cities. I have been impelled to lay the preceding before the profession in view of the extraordinary course which this "Journal," so professedly conservative of our interests, is taking in the matter. To our great surprise and indignation, in a recent issue of the "Journal" appeared the original call for the Convention. Not one of the signatures, to the best of our knowledge and belief, is that of a professional man; all, as far as we can ascertain, are quacks of the first water. There is not the slightest excuse for such a direct insult to us; I gave Mr. Smith the names of those graduates practising here, and informed him of the standing of those men whose signatures he had already obtained. We emphatically protest against being placed in this anomalous position by the so-called "U. S. Veterinary Journal"; we declare the Convention to be held in St. Louis, Mo., May 6, 1884, under the auspices of said "Journal," to be a direct insult to the profession at large, and the practitioners of St. Louis in particular; we refuse to believe that the proper way to advance the interests of the veterinary profession is to form a coalition with quackery; we absolutely deny the right of the "U. S. Veterinary Journal" to put aside the objections of graduates, and advance their private interests at the expense of our young and noble profession; we ask the professional papers to ventilate the matter thoroughly; and finally, we pledge ourselves to unflinchingly withstand any such infringement of our rights as is proposed by the commercial clique in question. We are few in number; our opponents are many. Let all thinking members of the profession give us their moral support. Vis unita fortior.

H. F. JAMES, V. S. (Ontario),
2627 Olive Street, St. Louis, Mo.

